# The Alberta Infant Motor Scale: A tool for the assessment of motor aspects of neurodevelopment in infancy and early childhood

**DOI:** 10.3389/fneur.2022.927502

**Published:** 2022-09-14

**Authors:** Małgorzata Eliks, Ewa Gajewska

**Affiliations:** ^1^Chair and Clinic of the Developmental Neurology, Poznan University of Medical Sciences, Poznań, Poland; ^2^Doctoral School, Poznan University of Medical Sciences, Poznań, Poland

**Keywords:** Alberta Infant Motor Scale (AIMS), infancy, motor development, childhood, scale

## Abstract

According to the recommendations of the American Academy of Pediatrics, the surveillance of motor development should accompany systematic appointments with medical professionals in infancy and early childhood. One of the standardized tools for evaluating motor development is the Alberta Infant Motor Scale (AIMS). This paper aims to present assumptions and psychometric properties of the AIMS, the methodology of assessment of an infant's performance with the AIMS, and research on the validation and standardization of the AIMS as well as the use of the scale as an outcome measure. We conducted a non-systematic literature review using three electronic databases: PubMed, Scopus, and Embase (from June 1992 to February 2022). We included original research with a full-text manuscript in English. No geographical restrictions were applied. The search terms “alberta infant motor scale” AND “reliability” OR “validity” and “alberta infant motor scale” AND “norms” OR “reference” OR “standardization” were used for literature review on the validation and standardization of the AIMS in other non-Canadian populations. This narrative review also focuses on how the AIMS is applied as an outcome measure in research by presenting studies on the AIMS conducted over the last decade. Our review found that the AIMS is widely used for both research and clinical purposes. The AIMS has been used as an outcome measure in both interventional and observational studies conducted on both neurotypical infants and those with conditions affecting motor development. The advantages of the scale are its infant-friendliness, time duration of the examination, and relative ease of application for an examiner. The scale has been validated and standardized in many countries.

## Introduction

Surveillance of early motor development can facilitate the detection of motor delays or disturbances (i.e., in postural and/or movement control, abnormal movement patterns, and muscle tone) leading to early interventions aimed at preventing future structural or functional disorders from developing (1). According to recent recommendations from the American Academy of Pediatrics, pediatricians should routinely monitor early development and conduct standardized developmental screenings at 9, 18, and 30 months of age (1).

One of the standardized tools for evaluating motor development is the Alberta Infant Motor Scale (AIMS). The AIMS considers both quantitative (e.g., occurrences of motor skills) and qualitative characteristics (e.g., the manner of motor performance). The scale may be applied by healthcare professionals involved in infants' development screening up to 18 months of age for either clinical or research purposes. The AIMS has been implemented in many populations. This review is centered on the AIMS because it is highly applicable and has excellent psychometric properties (e.g., reliability and validity). We aim to investigate the psychometric properties of the AIMS and how infants perform during an assessment and investigate the validity and standardization of the tool as an outcome measure in many different research contexts.

Although this paper is not a systematic review, methodological details pertaining to how the search was conducted are described below. We conducted a literature review using three electronic databases: PubMed, Scopus, and Embase (from June 1992 to February 2022). We included original research with a full-text manuscript in English. No geographical restrictions were applied. The search terms “alberta infant motor scale” AND “reliability” OR “validity” and “alberta infant motor scale” AND “norms” OR “reference” OR “standardization” were used to identify relevant articles pertaining to the use of the AIMS in non-Canadian populations. Studies using the AIMS were excluded from the review if they were used to validate other tools or scales. To provide the full coverage of the literature, additional searches of the reference lists of included studies were performed. This paper is constructed as a narrative review detailing also how the AIMS has been used as an outcome measure in different research applications. We included papers ranging from systematic reviews and randomized controlled trials (RCTs) to observational studies published in the past decade.

## The Alberta Infant Motor Scale

The Alberta Infant Motor Scale (AIMS) is a standardized tool used to assess gross motor development from birth (40 weeks conception) through independent walking (18 months of age) (2). The AIMS was created in the early 1990's by Martha C. Piper and Johanna Darrah from the University of Alberta, Canada (2). The tool consists of both the scoresheet and manual (2, 3).

The AIMS is designed to (1) identify infants with motor delay, (2) provide information to medical professionals and parents on the motor achievements of the infant, both currently developing activities and those not observed in the infant's repertoire, (3) evaluate motor performance over time or before and after an intervention, and (4) function as a research tool assessing the efficacy of rehabilitation programs for infants with motor delays (4). The AIMS monitors the motor skill achievement of infants: (1) with typical motor development and no medical concerns, (2) with no predisposing factors in pre-, peri-, or neonatal medical histories, but identified as having suspect development in routine medical examinations, (3) considered to be at risk for the developmental delay because of their medical history (e.g., those born preterm), and (4) infants with a diagnosis, e.g., Down syndrome (3). The AIMS was created with assumptions of the dynamic systems theory of motor development, which encompasses ontogenetic, environmental, and task factors in addition to incorporating some elements of the traditional neuromaturational model (2). Historically, the first theory of motor development was the neuromaturational model by Gesell, McGraw, and Shirley, which assumes that progress in motor skills and body control derives from the maturation of the central nervous system (CNS) and processes like myelinization, synaptogenesis, and gradual inhibition of the lower subcortical nuclei of the brain by the cerebral cortex (2). This model incorporates tenets such as (1) a progression of movement from primitive global movement reflex patterns to voluntary, controlled movement, (2) a cephalocaudal, (3) a proximal to the distal direction of motor development progression, and (4) a consistent sequence and rate of motor development (2). According to the dynamic systems theory, motor development is determined by the cooperation of some subsystems (the maturation of the CNS being one of them), and proceeds dynamically in the adaptations to factors or depending on an infant or environment (2). This model incorporates a correlation between neurobiological, biomechanical, psychological, familial, and environmental conditions (2). The subsystems include postural control, muscle strength, body weight, an infant's mood and motivation, environmental condition, and task requirements (2).

## Using the AIMS to assess motor development

The scoresheet of the AIMS consists of 58 items at four positions (21 in prone, 9 in supine, 12 in sitting, and 16 in standing) (4). The components assessed for each item are based on three elements: weight-bearing, posture, and antigravity movements (4). An example may be the item “swimming,” in which weight-bearing involves weight on the abdomen, posture is described as symmetrical with adducted scapulae, abducted and externally rotated arms, abducted and extended legs, extended lumbar spine, and antigravity movement involves a raising head and arms or legs, or both from the surface and active extensor pattern (2). A drawing with the infant's position and a short description accompany every item, and the manual covers their full description (3). Though examiners are evaluating spontaneous motor performance, they are allowed to interact with infants to encourage them to demonstrate their skills. The examination is performed on a table or a mat (for infants older than 4 months) (3). A parent/caregiver should be present during the assessment to provide comfort for an infant (3). However, some items (in sitting or standing) require certain positioning or physical prompting by an examiner (3). The last and the most mature items are identified in every position—these two items constitute the developmental “window” and then score every item in the “window” as “observed” or “not observed” (3). Each item below the least mature is treated as “observed.” The scoring is a dichotomous choice for each item—“observed” (1 point) or “not observed” (0 points) (3). To receive 1 point, every element of an item has to be accomplished (3). The sum of all the items in every position gives the total raw score, which may be converted to percentile ranks (with 1-month-age group intervals) (3). The duration of the assessment is about 20–30 min and may be performed with direct observation or assessment of video recordings (3, 5). For diagnostic use, the cut-off scores on the percentile ranks for atypical development are identified as the 10th percentile at 4 months of age and the 5th percentile at 8 months of age (3). The AIMS is most sensitive when performed on infants between 4 and 12 months old. While the authors of the AIMS recommend that healthcare professionals with knowledge of infant development and experience in observational evaluation of motor performance perform the assessment (3), Snyder et al. found that the scale may be also approachable for novice examiners (6). The intra-class correlation (ICC) between groups of experienced and novice examiners was scored at 0.98 in the assessment of infants younger than 10 months (6). Nonetheless in the evaluation of infants older than 10 months, significant differences between raters were found (6). The authors found that the varied motor performance of older infants is better assessed by more experienced raters (6).

## The psychometric properties of the AIMS

The AIMS is used worldwide for developmental assessments due to its distinctive psychometric values. The reliability and validity studies of the original scale were performed in a group of healthy Canadian infants; 253 infants were assessed for test–retest and interrater reliabilities, and 120 infants were involved in the assessment of the concurrent validity with motor scales of the Peabody Developmental Motor Scales (PDSM) and the Bayley Scales of Infant and Toddler Development (BSID) (4). The test–retest and inter-rater reliability were set at 0.99 (4). The concurrent validity was defined as 0.97 with PDSM and 0.98 with BSID (4). The normative values were established in the group of 2,202 infants from Alberta and were expressed as mean, standard deviation (SD), and percentile rank for total score in every month of age, as well as time norms for achievement of every item (4). The tables with reference norms and figures with percentile ranks are available in the manual (3). The re-evaluation of the scale in 2014 suggests that the normative values in the Canadian population remained stable over time (7).

Unlike the BSID or the PDMS, the AIMS considers both quantitative and qualitative aspects of developmental assessment—it includes well-known motor achievements, e.g., pivoting, rolling, reciprocal creeping, sitting, or walking alone with a meticulous description of their proper performance (4).

It is worth highlighting that the AIMS considers key gross motor milestones as stated by the World Health Organization (WHO) (sitting without support, standing with assistance, hands-and-knees crawling, walking with assistance, standing alone, and walking alone) (8).

## Research on the validation and standardization of the AIMS in other populations

Developmental assessments should be based on evidence-based medicine and performed with standardized tools—culturally adapted and validated if administered in languages, cultures, and countries other than the original (9). This process includes two forward translations of the tool into the new language, synthesis of the translations together, two back translations, and consolidation within the expert committee to receive semantic, idiomatic, experimental, and conceptual equivalences, and then testing the prefinal version and measuring reliability and validity of the final version (9).

The first studies on the reliability and validity of the AIMS in non-Canadian contexts were performed in Taiwan (10) and Japan (11), but only in premature infants in the Taiwanese population (10). The AIMS has been translated into Portuguese (12), Spanish (13), Chinese (14), Thai (15), Serbian (16), and Korean (17). The validation of these language versions varied in terms of the sample size (from 30 to 259 infants), the character of the group (full-term or full-term and preterm infants, healthy or at developmental delay risk) as well as the methodology of the study (number of raters, referential diagnostic tool for the concurrent validation study). The reliability (both intra- and inter-rater) and concurrent validity studies were performed in Portuguese (12), Spanish (13), Chinese (14), and Thai (15) versions. For the Serbian and Korean versions, only reliability values were investigated. The results of the existing research are presented in Table 1.

**Table 1:** The studies on the reliability and validity of the AIMS.

**References, country**	**Population**	**Translation**	**Intrarater reliability**	**Number of raters in the interrater reliability study**	**Interrater reliability**	**Referential diagnostic tool for the concurrent validation study**	**Concurrent validity**
Piper et al. (4), Canada	506 normal infants (aged 0–18 months)	original version	0.99 (sample of 233 infants)	2	0.99 (sample of 221 infants)	BSID I, PDMS-1, motor scales	0.98 with BSID I, 0.97 with PDMS-1 (sample of 120 infants)
Jeng et al. (10), Taiwan	86 preterm infants (aged 0–18 months)	no	0.85–0.99 (sample of 45 infants)	3	0.73–0.98 (sample of 45 infants)	BSID II motor scale	0.78–0.90 (sample of 41 infants at the age of 6 and 12 months)
Uesugi et al. (11), Japan	40 healthy infants (aged 22 days to 16- months 27 days)	no	0.86–0.93	6	≥0.94	Kyoto Scale of Physiological Development	0.97 0.98
Valentini and Saccani (12), Brazil	766 preterm and full-term infants, (aged 0–18 months)	Portuguese	0.91–0.99 (sample of 259 infants)	3	0.86–0.99 (sample of 259 infants)	Child Behavior Development Scale	0.34 (sample of 40 infants)
Morales-Montforte et al. (13), Spain	50 infants at risk or at diagnosis of developmental delay, [aged 0–18 months)]	Spanish	0.94–1.00	2	0.95–1.00	BSID III	0.97 (0–3 months), 0.69 (4–8 months), 0.96 (9–18 months) (sample of 25 infants)
Wang et al. (14), China	50 infants at high risk, (aged 0–9 months)	Chinese	0.81–0.99	3	0.98–0.99	PDMS-2	0.75–0.97 (sample of 47 infants)
Aimsamrarn et al. (15), Thailand	30 full-term healthy infants, (aged 0–18 months)	Thai	0.98–0.99	3	0.98	BSID III- scale motor	0.97
Lackovic et al. (16), Serbia	60 full-term infants at risk (aged 0–14 months)	Serbian	0.65–0.99	2	0.65–0.99		
Ko and Lim (2022), Korean (17)	70 pre-and full-term healthy infants (aged 0–18 months)	Korean	0.73–1.00	6	0.80–1.00		

AIMS, Alberta Infant Motor Scale.

PDMS, Peabody Developmental Motor Scales.

BSID, Bayley Scales of Infant Development.

The original version of the AIMS was developed in Canada with normative scores reflecting a Canadian population of infants. Studies comparing the Canadian normative AIMS values to other populations have identified several differences concerning motor development (Table 2). The research on Flemish and Dutch populations noted lower overall scores in these samples (18, 21, 23, 25). While the Thai study found significantly decreased scores in the first 3 months, infants aged 7–<8 months, 11–<12 months, and 13–14 months had considerably higher scores relative to Canadian norms (24). Similarly, lower AIMS scores in Turkish infants aged 0–1 and 2–3 months relative to Canadian norms were thought to possibly stem from cultural differences, such as swaddling of younger infants, which could limit early antigravitational movements and head control (26). In contrast, research into the use of the AIMS in the Brazilian context is mixed. While Gontijo et al. found no difference in the AIMS scores relative to Canadian norms (20), Saccani et al. found lower scores in Brazilian infants aged 0–15 months (22). Overall, only AIMS scores in Greek infants were consistent with the original Canadian norms (19). The differences in the AIMS scores between populations advocate for a need to validate and standardize the scale across various ethnic and cultural contexts.

**Table 2:** The studies on the standardization of the AIMS in other populations than the original.

**References, country**	**Population**	**Number of raters**	**Intrarater reliability**	**Interrater reliability**	**Translated version of the AIMS**	**The comparison to Canadian norms**
Fleuren et al. (18), The Netherlands	100 infants (aged 0–12 months)	2	___	0.99 (sample of 8 infants)	No	Overall lower scores
Syrengelas et al. (19), Greece	1,068 full-term infants, (aged 7 days to 19 months)	3	___	___	No	No differences in all age samples between both populations
Gontijo et al. (20), Brazil	660 full term infants, (aged 0–18 months)	3	___	0.93–0.97 (sample of 10 infants)	Portuguese	No differences in all age samples between both populations
De Kegel et al. (21), Belgium	270 infants, (aged 9 days−18 months 8 days)	4	___	0.99 (sample of 18 infants)	No	Overall lower scores
Saccani et al. (22), Brazil	1,455, full- and preterm (aged 0-18 months)	3	___	0.86–0.99	Portuguese	Lower scores
Suir et al. (23), The Netherlands	499 developing typically infants full-and preterm(aged 2 weeks to 19 months)	2–4	___	0.98 (sample of 8 infants)	No	Lower scores
Tupsila et al. (24), Thailand	574 full-term infants (aged 15 days to 14 months)	3	0.97–0.99 (sample of 25 infants)	0.99 (sample of 25 infants)	Thai	No differences in samples between 4 to 6 months, 8 to 10 months, and 12–13 months
van Iersel et al. (25), The Netherlands	1,697 pre-and full-term infants, (aged 2–18 months)	>2	___	___	No	Overall lower scores
Kepenek-Varol et al. (26), Turkey	411 full-term infants, (aged 5 days-18 months)	1	___	___	No	No differences, except the samples aged 0–1 and 2–3 months

AIMS, Alberta Infant Motor Scale.

## The Alberta Infant Motor Scale as the outcome measure in studies on motor development

The AIMS has been used for both clinical and research purposes. The scale has been used as an outcome measure for not just healthy infants, but those with a range of disorders or conditions affecting motor development.

## Preterm infants

Fuentefria et al. conducted a systematic review assessing the motor development of premature infants with the AIMS based on 23 articles published between 2006 and 2015 (27). The ages of the infants assessed in the studies varied from 0 to 18 months (27). Significant differences in motor development of pre- and full-term infants were identified, with preterm infants characterized by lower gross AIMS scores (27). Fuentefria et al. conclude that the AIMS is an appropriate tool for following up on motor development in infants, as well as identifying atypical development in infants born preterm (27).

## Structural brain disorders

Other cohorts analyzed with the AIMS were infants with CNS disorders acquired perinatally due to conditions like hypoxic-ischemic encephalopathy (HIE) and cystic periventricular leukomalacia (PVL), or prenatally due to conditions like Zika congenital syndrome.

Wu et al. conducted a phase-II clinical trial investigating the effectiveness of multiple doses of erythropoietin (EPO) administered with hypothermia on neuroradiographic and neurodevelopment of 24 newborns with HIE (28). They used the AIMS as an outcome measure for assessing motor performance when infants were 12 months old (28). Their results suggest that the treatment with EPO resulted in significantly better movement behavior in comparison to the control group (28).

A subsequent study on the influence of perinatal HIE on motor outcomes with the AIMS was performed by Procianoy et al. (29). The authors followed up infants born >35 weeks with moderate to severe encephalopathy (2 or 3 stages in clinical classification by Sarnat and Sarnat) and evidence of perinatal asphyxia who underwent whole-body hypothermia before 6 h of life (29, 30). Thirty-four neonates were imaged at 18 ± 8.4 days of life, with MRI results in 19 patients revealing posterior Limb Internal Capsule (PLIC) signs, lesions in the thalamus, the basal ganglia, the white matter, and the cortical areas (29). All infants had neurodevelopmental follow-ups between 12 and 18 months of age by trained professionals blinded to MRI findings (29). Results indicated a significant association between delay in motor development as determined by scores on the AIMS and the presence of severe encephalopathy with PLIC sign, the thalamus and basal ganglia, the white matter, and the cortical lesions identified by MRI performed in the neonatal period (29). Wang et al. compared motor abilities of preterm infants (born <27 weeks old) with very low birth weight born with and without PVL (observed *via* cranial ultrasound) after HIE relative to healthy controls (31). They assessed participants with the AIMS at three-time points of corrected age; at 6, 12, and 18 months (31). Study results suggest that cystic PVL was the most determining factor predicting motor development in preterm infants (31). Infants with PVL had significantly overall lower scores in every assessment relative to those without PVL and controls (31). At the 18-month assessment, no significant difference in AIMS scores was found between preterm infants without PVL and controls (31).

The studies described above indicate an association between findings of radiological diagnostic and motor performance in these groups of patients. Having radiological assessments may be substantial in planning early intervention.

Margues et al. investigated motor trajectories (at 6, 12, and 18 months of age) of 39 Brazilian infants affected by congenital Zika syndrome virus (32). The study was performed after the Zika virus outbreak between 2015 and 2016. The study results demonstrated that AIMS scores for infants affected by congenital Zika virus syndrome had broadly slowed motor development (32). In children affected by congenital Zika, AIMS scores at 6, 12, and 18 months corresponded to typical AIMS scores of controls aged 2–3 months, 3–4 months, and 4–5 months of age, respectively (32). Moreover, 35 of 39 participants met the criteria for the diagnosis of cerebral palsy (CP) (32). The current prevalence of CP in children infected by the Zika virus prenatally is about 80–100 % (33). CP related to Zika virus infection is thought to stem from prenatal brain abnormalities (microcephaly is the most prominent one) (33). The clinical manifestation of CP in this group has been characterized as an occurrence of hypertonia, spasticity, and hyperreflexia related to pyramidal tract involvement during the first months of life and extrapyramidal signs, such as dystonia and dyskinetic movements observed in the second year (33).

## Congenital cardiac disorders

Currently, only two studies have analyzed the motor development of infants with congenital cardiac disorders using the AIMS (34, 35). Studying neurodevelopment in this population is important as typical development may be negatively impacted due to factors like abnormal fetal blood flow, or negatively affected by medical interventions such as cardiopulmonary bypass, respiratory support, or perioperative hemodynamic instability (36).

Rajantie et al. compared motor development of infants with hypoplastic left heart syndrome (HLHS, *n*=23) and other types of univentricular heart conditions (UVH, *n* = 13) treated surgically relative to 47 healthy controls aged 16–52 weeks old (34). At 4 months, patients with HLHS or UVH had significantly lower AIMS scores than controls in the prone and supine positions (34). Additionally, infants with UVH presented lower scores than controls in the AIMS sitting items (34). At 12 months old, patients with HLHS had significantly lower scores in all AIMS positions, while infants with UVH had lower scores only in the prone and standing (34). Uzark et al. used the AIMS to help assess the concurrent validity of the Congenital Heart Assessment of Sensory and Motor Status (CHASM) tool designed to assess an infant's state post-cardiac surgery (35). They used both the AIMS and CHASM to assess the performance of 4- to 10-month-old infants, who underwent surgical cardiac interventions including tetralogy of Fallot repair, ventricular septal defect (VSD), complete atrioventricular septal defect repair, arterial switch operation ±VSD repair, Hemi-Fontan/bidirectional Glenn or other complex operations (35). The authors also assessed whether participants required physical therapy following a period of immobility, supine positioning, lying-in, sedation, and/or analgesic treatment due to their cardiac surgeries (35). For infants who underwent surgical cardiac interventions, main disturbances in motor development involved (1) forearm support and lifting head to 45 degrees in the prone, (2) holding head erect in the midline in a supported sitting position, and (3) sitting with support propped on extended arms (35). This observation may help inform physical therapy management approaches for infants following surgical cardiac interventions.

## Genetic disorders

Motor development delay in infants and children with Down Syndrome (DS) is apparent. One of the scales applied in this population is the AIMS (37). In the study by Pereira et al., the detailed characteristics of the acquisition of gross motor skills within the first year of life were investigated (38). A previous study by Tudella et al. suggested that relative to full-term controls, participants with DS present significantly lower AIMS scores in every position (39). However, the sequence of the acquisition of motor skills was the same in both groups (39). In further research, Pereira et al. analyzed timepoints of acquiring motor skills in prone, supine, sitting, and standing positions in 20 infants with DS and 25 typical controls aged 3–12 months (38). Participants were assessed monthly using the AIMS until they aged 12 months (38). All DS infants underwent physiotherapy based on the Bobath concept and Sensory Integration (38). In the prone position, the difference in developing items from the prone position (3rd item) to the four-point kneeling to sitting or half-sitting (16th item) was 1–3 months compared to controls (38). Only one DS infant completed the most advanced skill—the reciprocal creeping with lumbar spine flattening (21st item) by 12 months of age (38). In the supine position, the difference in acquisition skills from the supine lying with head at midline and arms along the body (third item) to the hand to feet (seventh item) was 1 month relative to typical infants (38). Interestingly, by 8 months old, all DS participants could move from the rolling supine to prone position with rotation (ninth item). In sitting, a delay in developing the sitting with support (first item) to the sitting without arm support and ability to shift posture (12th item) was 1–4 months regarding controls (38). The unsustained sitting without arm support (6th item) was acquired by every DS infant (38). Infants with DS were 2–3 months delayed in developing the supported standing [characterized by standing where their hips are aligned with their shoulders, active control of their trunk, and variable movements of legs (third item) to the half-kneeling position (eighth item)] (38). In addition, none of the infants with DS were able to stand independently at 12 months old (38). The results of this study have meaningful clinical implications. The acquisition of skills in the prone position was more challenging than when infants were supine (38). Vertical orientation, that is, sitting and standing activities, which involve antigravity activation of muscles in the neck, trunk, and upper and lower limbs muscles is limited in infants with DS (38). The results suggest that early therapeutic motor intervention should comprise the adoption of positions and antigravity movements, facilitation of concentric and eccentric contraction of trunk muscles, and transferring positions for space orientation to enable the acquisition of further motor skills (38).

Reus et al. studied growth hormone (GH) therapy combined with child-specific motor training on motor development in infants and children with Prader-Willi syndrome (PWS) (40). Motor developmental delay in PWS is thought to be related to muscular hypotonia, weakness, abnormally high fat-to-muscle ratio, and cognitive retardation (40). For 2 years, the authors followed 22 newly diagnosed children with PWS (mean age of 12.9 months at study onset) divided into two groups: (1) 10 participants administered GH for 24 months, and (2) 10 participants administered GH after 6 months of a control period (40). Two individuals, whose parents refused hormonal therapy, were additionally included in the control group (40). Every participant attended an individualized physical training program, and motor performance was assessed every 3 months (40). The results of motor development measured in the AIMS revealed that a higher initial level of motor development was associated with persistently better performance over time relative to a lower developmental level at the beginning of the trial (40). In addition, infants who received GH treatment at study onset reached the end of the AIMS earlier than infants who had a 6-month control period where GH was not administered (40). While age and GH had significant interaction effects on the AIMS, the intake of GH was found to have a significant positive effect on motor development in infants with PWS (40). For infants with PWS, the final items on the AIMS (15th item—walking, 16th item—squatting) were achieved at a mean age of 27.5 months and 38.3 months, respectively (40). In contrast, 90% of typically developing infants acquired the ability to walk and squat between 14 and 15 months old (3). Of note, however, the AIMS was used to assess motor development in infants >18 months old—something the tool was not designed for.

Huggins et al. followed 20 infants and children (aged 6–21 months) newly diagnosed with late-onset Pompe disease (LOPD) to determine a clinical phenotype for this group (41). While most LOPD infants had average or above-average scores on the AIMS, participants' scores were extremely variable and ranged between the 5th and 90th percentiles (41). The authors suggest that in further research on specific groups of patients, a detailed assessment of the musculoskeletal system should accompany the AIMS.

## Nonsynostotic plagiocephaly

The AIMS has also been used in populations with less severe developmental conditions commonly seen by pediatricians or pediatric physiotherapists such as positional plagiocephaly. Two RCT studies investigated the effectiveness of manual therapy in the management of nonsynostotic (positional) plagiocephaly (42, 43). Cabrera-Martos et al. analyzed the efficacy of manual therapy in infants with severe (type 4 or 5 on the Argenta scale) plagiocephaly on the reduction of head asymmetry, duration of treatment, and motor development (42). The study included 28 infants between 4 and 8 months of age allocated into two arms: the control group that underwent standard treatment (proper positioning and an orthotic helmet), and an experimental group that received standard treatment in addition to manual therapy (45-min sessions/week) based on an osteopathic approach (42). The baseline assessment showed lower AIMS scores than expected in both groups. Despite both control and treatment arms having normal motor performance upon study discharge, the experimental group received a significantly shorter treatment duration (mean of 109.84 days relative to 148.65 days in controls) (42). The subsequent research by Pastor-Pons et al. investigated the efficacy of pediatric integrative manual therapy in 34 infants with positional plagiocephaly (with a minimal difference of 5 mm between cranial diagonal diameters) younger than 28 weeks of age (43). The control group received an evidence-based educational program including exercises to limit positional preference and advising parents on positioning, baby management, and care to stimulate (43). In addition to the standard treatment the control arm received, the experimental arm received additional manual therapy centered on mobilization techniques of the occiput, atlas, and axis (ten 20-min sessions once a week) based on the pediatric integrative manual therapy (PIMT) (43). After the intervention, a significantly higher increase in the cervical spine active rotation range of motion in the PIMT group was observed relative to the control group (43). Similar to Cabrera-Martos et al., Pastor-Pons' RCT found no difference in motor development between control and treatment arms at the trial conclusion (42, 43).

## Conclusion

The AIMS is a standardized tool for motor development that has been validated for use in infants aged 0–18 months old. The scale is widely used for both clinical and research purposes. The AIMS has been used as an outcome measure assessing motor performance in infants in both RCTs and interventional or observational studies. It has been used to assess motor development across many different infant populations including infants born preterm, infants with surgically treated congenital cardiac disorders, and infants with genetic or structural brain disorders. While the AIMS has been standardized and validated for use in South America, Asia, and both South and West Europe, no study to date has validated its use in central Europe. One of the main advantages of the AIMS in both clinical and research contexts over other tools assessing motor development is that it is very infant-friendly, does not take long to administer, and the relative ease of administration. Overall, the AIMS is a tool well-suited for assessing early motor development in infants across many geographical and cultural contexts.

## Author contributions

ME: research concept and design, collection of data, data analysis and interpretation, writing the article, and final approval of the article. EG: research concept and design, critical revision of the article, and final approval of the article. Both authors contributed to the article and approved the submitted version.

## Funding

This paper was partially financed from the large research grant from statutory funding for young researchers-doctoral students for 2021. Poznan University of Medical Sciences, grant number [SDUM-GB9/03/21].

## Conflict of interest

The authors declare that the research was conducted in the absence of any commercial or financial relationships that could be construed as a potential conflict of interest.

## Publisher's note

All claims expressed in this article are solely those of the authors and do not necessarily represent those of their affiliated organizations, or those of the publisher, the editors and the reviewers. Any product that may be evaluated in this article, or claim that may be made by its manufacturer, is not guaranteed or endorsed by the publisher.
